# TRPV3 in Drug Development

**DOI:** 10.3390/ph9030055

**Published:** 2016-09-09

**Authors:** Lisa M. Broad, Adrian J. Mogg, Elizabeth Eberle, Marcia Tolley, Dominic L. Li, Kelly L. Knopp

**Affiliations:** 1 Lilly Research Centre, Eli Lilly and Company Ltd., Erl Wood Manor, Windlesham, Surrey GU20 6PH, UK; mogg_adrian@lilly.com; 2 Covance Greenfield Laboratories, Greenfield, Indianapolis, IN 46140, USA; eberle_elizabeth_lutz@network.lilly.com (E.E.); tolley_marcia_k@network.lilly.com (M.T.); 3 Lilly Research Laboratories, Eli Lilly and Company Inc., Indianapolis, IN 46285, USA; dominic.li5702@gmail.com (D.L.L.); knopp_kelly_l@lilly.com (K.L.K.)

**Keywords:** TRPV3, keratinocytes, itch, pain, Olmsted syndrome

## Abstract

Transient receptor potential vanilloid 3 (TRPV3) is a member of the TRP (Transient Receptor Potential) super-family. It is a relatively underexplored member of the thermo-TRP sub-family (Figure 1), however, genetic mutations and use of gene knock-outs and selective pharmacological tools are helping to provide insights into its role and therapeutic potential. TRPV3 is highly expressed in skin, where it is implicated in skin physiology and pathophysiology, thermo-sensing and nociception. Gain of function TRPV3 mutations in rodent and man have enabled the role of TRPV3 in skin health and disease to be particularly well defined. Pre-clinical studies provide some rationale to support development of TRPV3 antagonists for therapeutic application for the treatment of inflammatory skin conditions, itch and pain. However, to date, only one compound directed towards block of the TRPV3 receptor (GRC15300) has progressed into clinical trials. Currently, there are no known clinical trials in progress employing a TRPV3 antagonist.

## 1. Introduction

TRPV3 is a non-selective cation channel, displaying relatively high permeability to calcium. It was first cloned in 2002 and displays ~30%–40% sequence homology with other TRPV channels.

**Figure 2:**
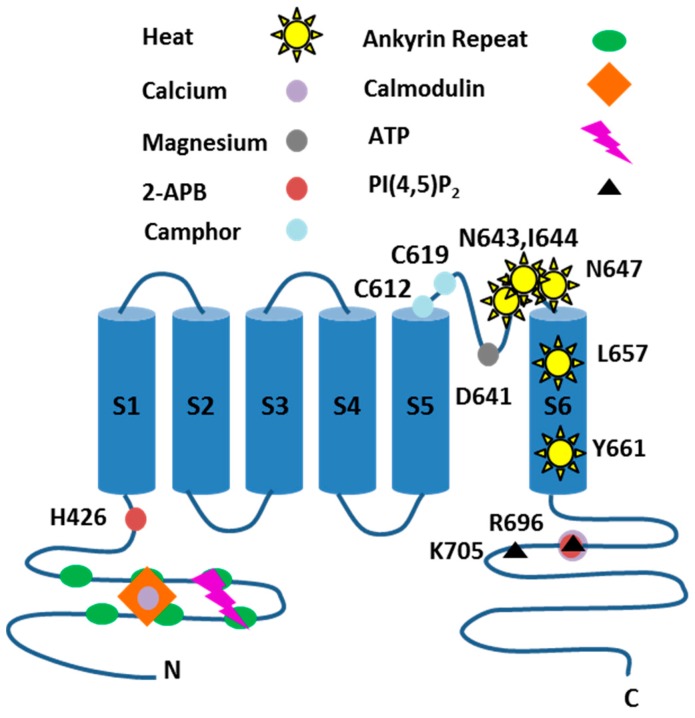
Membrane topology of TRPV3. Residues involved in heat activation (N643, I644, N647, L658 and Y661), activation by 2-APB (H426 and R696), Camphor (C612 and C619) and modulation by phosphatidylinositol 4,5-bisphosphate (PI(4,5)P_2_; R696 and K705), ATP (K169 and K174), Mg^2+^ (D641) and Ca^2+^ (R696) are highlighted, in addition to the location of ankyrin repeats [7,8,9,10,11,12,13].

Other features in common include the predicted protein structure encompassing six transmembrane spanning domains (1–6), a pore-forming loop between domains 5–6, cytoplasmic amino and carboxyl termini, ankyrin repeats and coiled-coil domains at the amino terminus and several putative phosphorylation sites [1,2,3] (see Figure 2). The channel is formed from four subunits, with homo-tetrameric TRPV3 receptors being commonly studied. Hetero-tetrameric channels containing TRPV3 and TRPV1, for example, are reported to be able to form by some [3,4,5], but not all researchers [6]; however, the nature, role and expression of native TRPV3 containing heteromers is far from clear.

**Figure 3:**
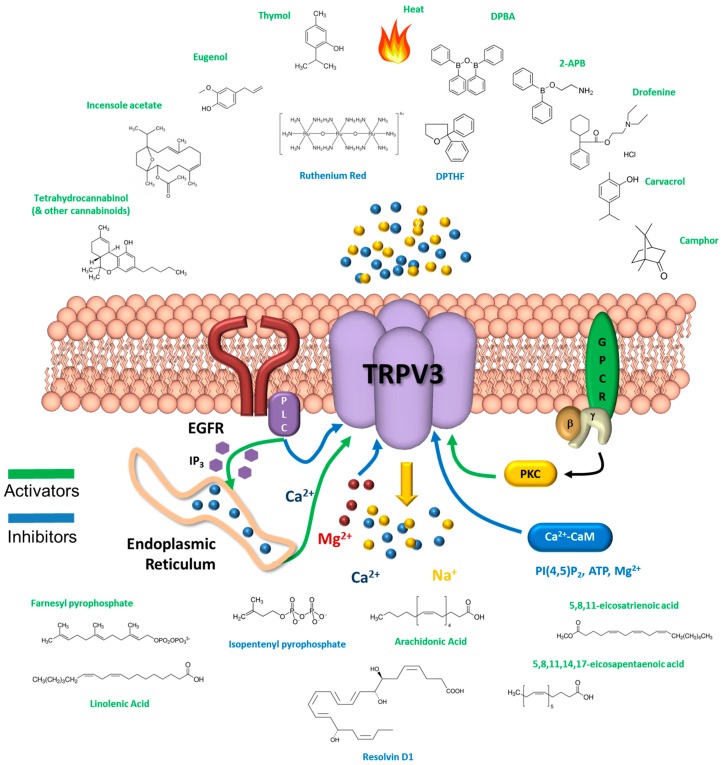
Activators, inhibitors and modulators of TRPV3. Quaternary structure of TRPV3 with compounds and signalling pathways known to activate, inhibit or modulate the receptor. See Introduction for references–additional references [23,24,25,26].

In common with other TRP channels, TRPV3 acts as a polymodal signal integrator. It is activated by numerous physical and chemical modulators and by posttranslational modifications, and the impact of individual stimuli is influenced by the presence of other stimuli (see Figure 3 for summary). TRPV3 is activated by innocuous temperature, with the threshold for channel opening at 31–39 °C, and activity is retained over temperatures extending into the noxious range [14]. Chemical agonists include spice extracts (such as camphor and carvacrol), synthetic agents (including 2-aminoethoxy diphenylborate, 2-APB) and endogenous ligand farnesyl pyrophosphate (FPP), [1,2,3,15,16]. Repeated exposure to heat or chemical agonists leads to sensitization of the receptor [8,17], through hysteresis of gating [14], while co-application of diverse stimuli, such as heat and chemical agonists, is synergistic [17,18]. Activation of G_q_ coupled GPCRs also sensitizes TRPV3 [19], with key components in the downstream signalling cascade including protein kinase C [20], Ca^2+^-calmodulin [8,10], phosphatidylinositol 4,5-bisphosphate (PI(4,5)P_2_) [11] and unsaturated fatty acids [18,20]. Other modulators include voltage [11], ATP [10], Mg^2+^ [12], and intracellular acidification [21,22].

The amino acid residues and regions underlying activation of TRPV3 by a number of stimuli have been mapped. Information relating to the mechanism of activation by heat [14,27,28], 2-APB [9] and camphor [13] is available. Likewise, residues involved in mediating modulation by Ca^2+^/Ca^2+^-calmodulin; [8,10]; Mg^2+^ [12]; pH [21] and voltage [11] have been defined (Figure 2, see Yang and Zhu for recent detailed review [29]).

## 2. Expression and Function of TRPV3

**Figure 4:**
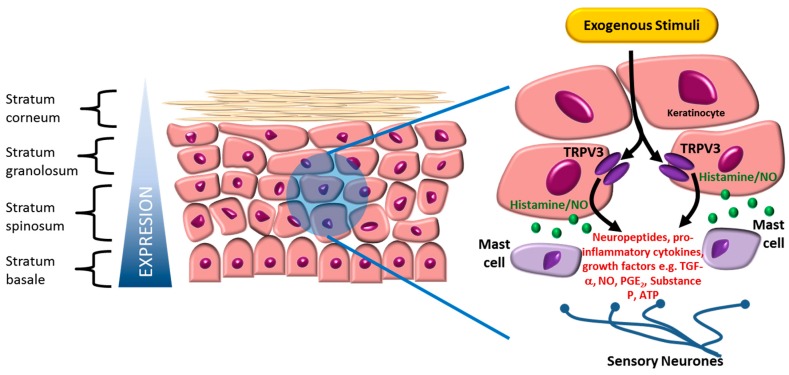
Summary of TRPV3 expression and function in epidermal keratinocytes. TRPV3 protein has been found throughout the epidermis and around hair follicles, with protein elevated under certain inflammatory skin conditions.

In contrast to TRPV1, TRPA1 and TRPM8, a direct role for TRPV3 in the peripheral nervous system is controversial, as expression in sensory neurons is not conserved across species, with minimal detection in rodents [1,2,3,30]. TRPV3 is however expressed in skin keratinocytes (Figure 4) across species [1,2,31] and in humans, TRPV3 gene expression in skin is the highest of the >50 tissues profiled in the GTEx project [32] (Supplementary Figure S1). TRPV3 expression is also reported in other epithelial cells including oral and nasal epithelium [19,33], distal colon [34] and cornea [35]. In oral epithelia, use of TRPV3 knock mice implicate this channel in wound healing [33]. Likewise, although TRPV3 gene expression in brain overall appears to be very low (Supplementary Figure S1), studies utilising TRPV3 knock-out mice are suggestive of a role for TRPV3 in regulating hippocampal synaptic plasticity [36] and incensole acetate, a reported TRPV3 agonist, broadly modulated brain activity in wild-type, but not TRPV3 knock-out mice [37]. Interestingly, there are several reports detailing TRPV1 channel expression on the endoplasmic reticulum of dorsal root ganglia and sarcoplasmic reticulum of skeletal muscle cells [38,39]. Indeed, it has recently been reported that there is functional expression of TRPV3 on the ER of mouse embryonic stem cells, with the channel having a putative role in controlling cell cycle and cellular proliferation [40]. These novel findings, if confirmed in native adult tissues, may need to be taken into account when developing compounds for TRPV3.

Until recently, reliance on non-selective agonist and antagonist tools, such as Ruthenium Red, has hampered progress in clearly defining a functional role for native TRPV3. For example, evidence for a role of TRPV3 in modulating the CNS dopaminergic system [41,42] and colonic [34] and corneal epithelia responses [35] is largely circumstantial for this very reason. Perhaps unsurprisingly, keratinocytes have been the most commonly utilized preparation for the study of native TRPV3 function by far, and provide the most compelling data package. Early studies in m308 keratinocytes identified whole cell currents, increases in intracellular calcium and release of IL-1α in response to application or co-application of known TRPV3 agonists and modulators, including eugenol, DPBA, camphor, 2-APB and arachidonic acid [19,20]. This work was later extended by Grubisha et al. (2014) [18], who confirmed that these responses could be blocked by a selective TRPV3 antagonist and by shRNA knock-down of TRPV3. In primary cultures of keratinocytes from wild-type mice, whole cell currents, increases in intracellular calcium or production of nitric-oxide were observed in response to TRPV3 agonists, or agonist combinations, including heat, 2-APB, carvacrol and camphor, these responses were sensitized on repeat application [12,17,43,44,45,46] and were absent in TRPV3 knock-out mice [17,45,46]. In transgenic mice over-expressing TRPV3 in skin keratinocytes, co-application of heat and 2-APB led to augmented TRPV3 mediated PGE_2_ release from keratinocytes [30]. In addition, application of 2-APB and carvacrol has been shown to promote TGF-α release from primary cultures of human keratinocytes [45], and in human keratinocyte HaCaT cells, 2-APB and acidic pH promoted whole cell currents blocked by Ruthenium Red [21].

Studies using keratinocytes have provided several insights for how TRPV3 regulates skin function and the function of neighbouring cells (Figure 4). A key protein interaction partner appears to be the epidermal growth factor receptor (EGFR). This receptor is proposed to form a signalling complex with TRPV3, whereby activation of the EGFR results in increased TRPV3 channel activity, stimulation of TGF-α release and epidermal homeostasis [45]. TRPV3 plays a role in maintenance of the skin barrier, as deletion of TRPV3 evokes deleterious changes in epidermal barrier structure. Hair morphogenesis is also disrupted in TRPV3 null mice [17,45]. In cultures of human outer root sheath keratinocytes, activation of TRPV3 induced membrane currents, elevated intracellular calcium, inhibited proliferation, induced apoptosis, inhibited hair shaft elongation and promoted premature hair follicle regression. These cellular effects, including inhibition of hair growth, were blocked by siRNA mediated TRPV3 knock-down [47]. α-Hydroxyl acids, commonly used in the cosmetic industry in chemical peels to promote exfoliation, have also been proposed to exert their effects through activation of keratinocyte TRPV3 [21]. Once again, the suggested mechanism is calcium influx through keratinocyte TRPV3 channels, leading to overload in intracellular calcium and cell death [21]. Of relevance to pain, TRPV3 mediated ATP release from keratinocytes has been shown to activate purinoceptors in dorsal root ganglion neurons [48], thereby influencing nociceptive function. Consistent with this, in transgenic mice over-expressing TRPV3 in skin keratinocytes, increased pain sensitivity was observed [30]. A number of related findings demonstrate that indirect modulation by keratinocytes can strongly influence pain signalling, for example in a recent study in TRPV1-knockout mice selectively expressing TRPV1 in keratinocytes, keratinocyte stimulation was sufficient to evoke acute nociception-related responses [49] and keratinocytes are also reported to induce neuronal excitability after nerve injury [50]. Of note, however, although TRPV3 null mice show disrupted responses to acute noxious heat and innocuous thermal sensation in thermotaxis endpoints [17], responses in other types of pain models are largely unaltered. In addition, the role of TRPV3 in innocuous temperature perception and acute thermal nociception appears to be somewhat dependent on gender and genetic background [46,51], for recent review, see Reference [52].

Several studies have reported alterations in TRPV3 mRNA and/or protein expression in pathological states, including in the skin of burns victims [53]; in rosacea, a chronic inflammatory skin condition [54]; in psoriasis [55] and in breast tissue biopsies from patients with breast pain [56]. Of note, aberrant TRPV3 function might be anticipated in some pathological states. For example, arachidonic acid, which is a strong potentiator of keratinocyte TRPV3, is present at high concentrations in psoriatic dermatitis (>100 μM; [57]) indicating that TRPV3 activity may be potentiated in psoriatic epidermis. Interestingly, a recent preliminary report describes a mechanistic link between TRPV3 activity in psoriasis through IL-1α and EGFR signalling [55].

## 3. Insights from Genetic Mutations

Adding further support to the role of TRPV3 in skin physiology and pathology, a TRPV3 gain-of-function G573S/C mutation was identified to underlie the spontaneously hairless, dermatitis phenotype of two rodent strains, DS-Nh mice and WBN/kob-HT rats, used as animal models of atopy [31,58,59]. Development of a TRPV3Gly573Ser transgenic mouse, recapitulated this phenotype, with significantly increased scratching behavior [58]. These transgenic mice also displayed augmented release of NGF in response to heat and significantly increased levels of serum chemokines and interleukins [58]. The increased release of pro-inflammatory and pro-nociceptive factors might be hypothesized to alter nociception, however data from exploration of the pain phenotype of TRPV3 gain-of-function rodents is only just emerging [60].

More recently, similar gain-of-function mutations in TRPV3, including but not limited to the Gly573 mutation, have been identified as the cause of Olmsted syndrome (OS) in humans (for review see [61]). OS is a rare keratinizing disorder characterized by marked thickening of the skin on the soles of the feet and palms of the hands, periorificial keratotic plaques, diffuse alopecia, and pruritus. In a few cases, atypical OS is reported with erythromelalgia. Pain and itching are variable in OS but are reported to be particularly severe in atypical OS patients with erythromelalgia, which leads to acute flares of hyperalgesia and some features of neuropathic pain.

## 4. TRPV3 Indications

Whilst activation of keratinocyte TRPV3 has been shown to mediate the release of pro-inflammatory and pro-nociceptive mediators and pruritogens, and the gain-of-function mutations implicate TRPV3 in skin conditions where a predominant feature is itch [62], there is a surprising lack of preclinical in vivo data exploring this aspect.

Selective TRPV3 antagonists have recently been introduced by Hydra Biosciences, Glenmark Pharmaceuticals and AbbVie (Section 5), and afford the opportunity to investigate the therapeutic potential of blockade of the TRPV3 channel.

**Figure 5:**
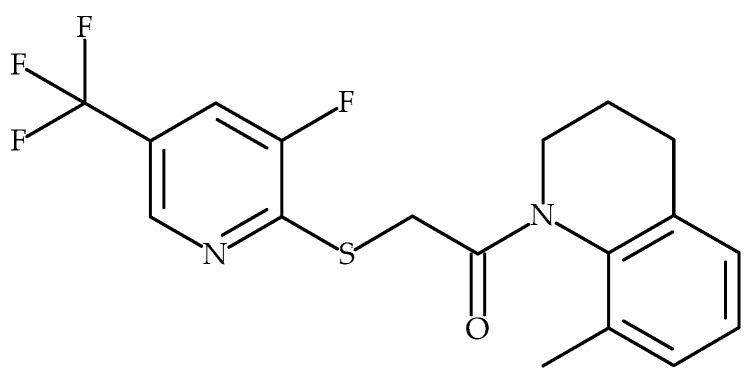
Structure of TRPV3 selective antagonist FTP-THQ [63].

**Figure 6:**
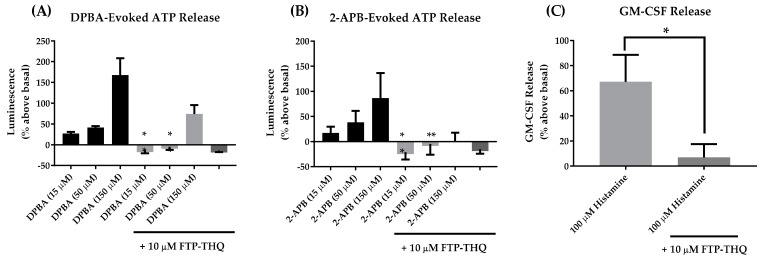
FTP-THQ [1-({[3-fluoro-5-(trifluoromethyl)pyridine-2-yl]sulfanyl}acetyl)-8-methyl-1,2,3,4-tetrahydroquinoline], a potent and selective TRPV3 receptor antagonist blocked TRPV3 mediated release of ATP (**A** & **B**) and GM-CSF (**C**) from mouse keratinocytes in vitro. Results are mean ± SEM of 3 independent experiments. Statistical significance was assessed using the paired Student’s *t*-test, * *p* < 0.05, ** *p* < 0.001.

We previously reported that Hydra’s FTP-THQ (Figure 5) was a potent and selective in vitro antagonist of recombinant and native TRPV3 receptors, having virtually no activity at TRPV1, TRPV4, TRPM8 and TRPA1 [18]. We have now assessed FTP-THQ in vitro in m308 keratinocytes and confirmed that it can prevent release of ATP and GM-CSF by concentrations of DPBA and 2-APB that are known to selectively activate TRPV3 in these cells ([18]; Figure 6).

**Figure 7:**
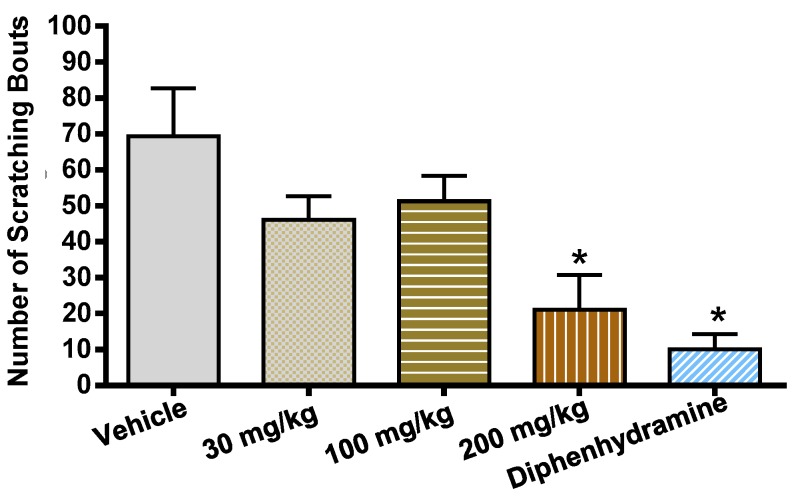
Effects of FTP-THQ on histamine-induced scratching behavior. Harlan CD-1 mice (*n* = 7–8/treatment group), 4–5 weeks old were acclimated to testing room for 1 h. FTP-THQ was administered at 30, 100, or 200 mg/kg i.p., 1 h prior to histamine, while diphenhyramine was administered at 20 mg/kg, 30 min prior to histamine. Animals were then placed inside a clear plexiglass chamber and the number of scratching bouts was scored for 20 min. Data were collected via Abacus software; one-way ANOVA with post-hoc Dunnett’s was used for analysis. * *p* < 0.05 vs. vehicle control.

FTP-THQ also has appropriate pharmacokinetic properties to assess its profile in vivo with an in vitro IC_50_ of 117 nM at the rat recombinant receptor and 186 nM at the mouse native receptor [18], and a Brain/Plasma ratio of approximately 7. In mice, after intraperitoneal administration, it dose-dependently blocked histamine-induced itch (Figure 7) with unbound exposure in brain (152 nM) consistent with the in vitro potency value, while the plasma levels where significantly less (37 nM). These data suggest TRPV3 can be pharmacologically modulated in a manner that is consistent with the gain-of-function mutations described in Table 1.

From the pain perspective, the genetic data are less compelling, but small molecule manipulation of the receptor with multiple compounds is suggestive of a role in nociceptive processing. For recent reviews, see References [64,65,66]. The TRPV3 agonist FPP is reported to elicit pain behaviors after intraplantar injection into inflamed animals [16]. The same group has also reported that 17(R)-resolvin D, an endogenous TRPV3 antagonist, is anti-nociceptive in acute and inflammatory pain states [24]. Complementarily, tool antagonist molecules from Hydra, Glenmark and AbbVie described below have attenuated pain behaviors in a number of pre-clinical pain models, including primarily carrageenan and Complete Freund’s Adjuvant-induced thermal and/or tactile hypersensitivity, but also in nerve ligation models [63,67,68].

**Figure 8:**
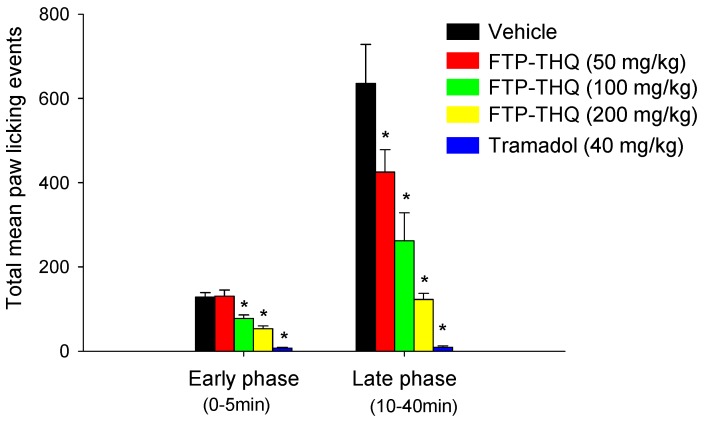
Effects of FTP-THQ on formalin-induced nocifensive behavior. Harlan Sprague Dawley Rats (*n* = 7–8/treatment group), were acclimated to the testing room for 1 h. FTP-THQ was administered at 50, 100, or 200 mg/kg i.p. 15 min prior to formalin, while the positive control tramadol was administered at 40 mg/kg i.p. 30 min prior to formalin. The animals were placed in Startle Behavior Chambers and behavior events (licking, guarding, flinching) binned in 5-min intervals and plotted as Early and Late Phase. Data were analyzed using 1-way ANOVA, and comparisons of drug treatment groups were compared with control groups using a post-hoc Dunnett’s comparison. * *p* < 0.05 vs. vehicle control.

We also evaluated FTP-THQ in a number of pre-clinical pain behavior models and replicated the previous findings in CFA-induced thermal hypersensitivity at the 200 mg/kg dose, and as exemplified in Figure 8, observed effects in the formalin-induced nocifensive responding assay. Interestingly, effects in this assay were observed at lower plasma/brain exposures. Confounding side effects, such as alterations in locomotor activity, were not observed, however, unlike previous reports (see [65]) we did observe a dose-dependent hypothermia with the antagonist. The thermo-sensing TRP channels have collectively been implicated as having the potential to alter normal thermal sensation and temperature regulation, due to findings with TRPV1 antagonists. Our data would suggest, at least in rodents, an alteration in body temperature should be closely monitored. It is unknown if these effects would translate into higher species. Taken together, the collective data indicate that potent and selective TRPV3 antagonists effectively attenuate scratching and pain behavior in pre-clinical animal models, and pharmacological blockade of this target may afford some therapeutic benefit.

By way of summary, Table 1 provides the potential therapeutic utility of TRPV3 modulators. However, as mentioned previously, no current clinical trials are ongoing.

## 5. TRPV3 Drug Development Overview

**Figure 9:**
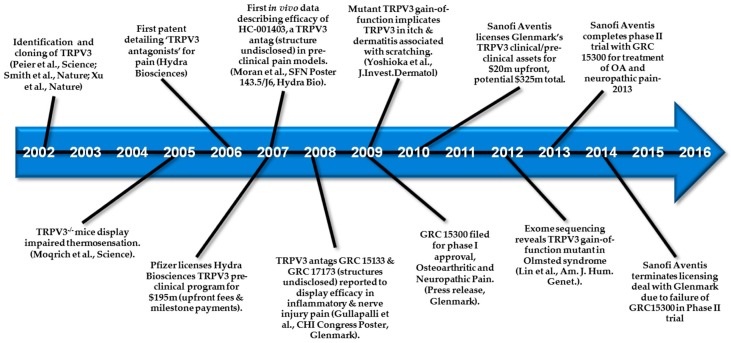
Timeline of major TRPV3 development activities.

**Figure 10:**
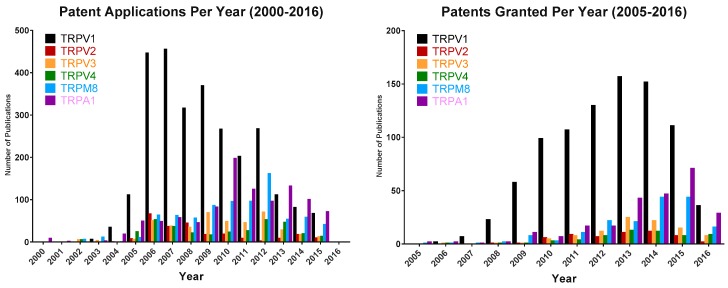
Number of patents applied for and granted per year for each of the thermo-TRPs compared to TRPV3 (orange bars).

Major milestones in the area of TRPV3 drug development are highlighted in Figure 9 and in comparison to TRPV1, the number of patents describing TRPV3 modulators has been modest (Figure 10) with Hydra Biosciences and Glenmark Pharmaceuticals being the main players.

### 5.1. Glenmark Pharmaceuticals Ltd.

**Figure 11:**
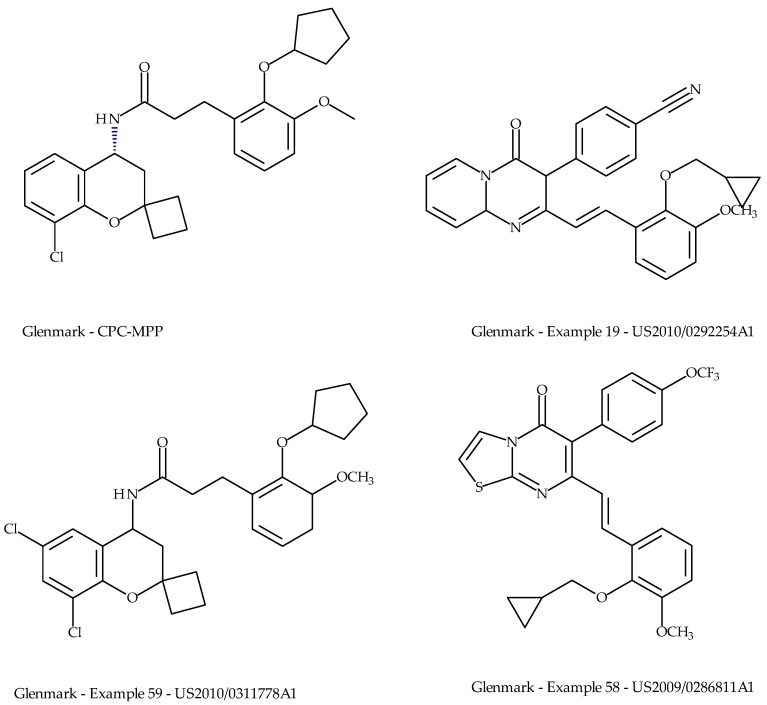
Examples of patented TRPV3 antagonists from Glenmark Pharmaceuticals.

Glenmark have patented a range of TRPV3 antagonists [80,81,82,83,84], Figure 11. In 2010, Glenmark entered into an out-licensing agreement with Sanofi Aventis and subsequently progressed their lead molecule (GRC15300, structure unknown) into the clinic for the treatment of osteoarthritic and neuropathic pain. In 2012, GRC15300 entered into Phase II trials for treatment of neuropathic pain; however, by the end of 2013, these trials had been discontinued. The Sanofi-Glenmark agreement was terminated in 2014 and since that date, no further development has been reported.

### 5.2. Hydra Biosciences Inc.

**Figure 12:**
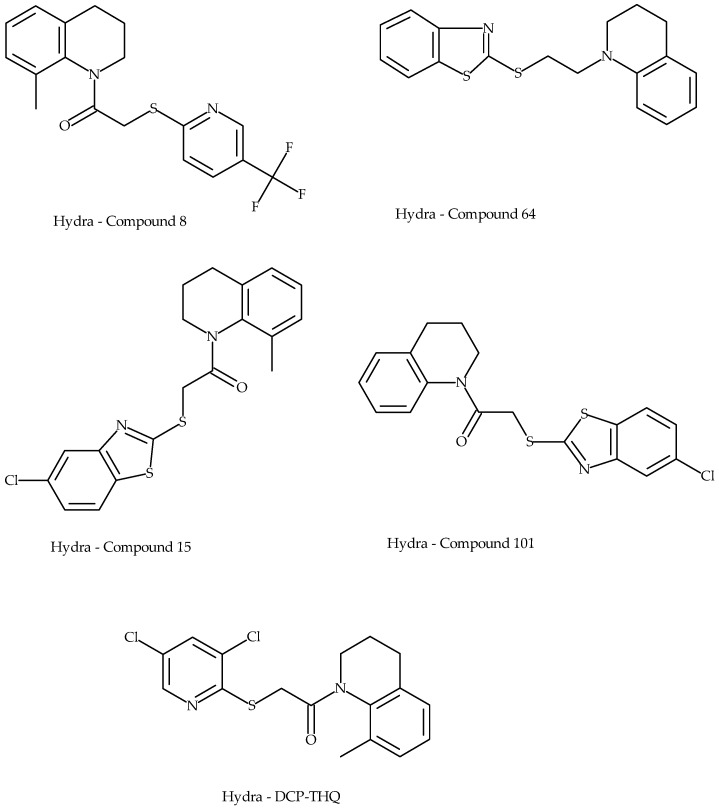
Representatives from Hydra Biosciences patented TRPV3 antagonist series.

Hydra Biosciences have also published several patents and publications on TRPV3 antagonists. In 2007, they entered into a collaboration with Pfizer to develop TRPV3 antagonists for pain. The company web page currently reports an active TRPV3 program directed toward dermatological disorders. In the published patents, there are descriptions of two compounds (Compound 15 and Compound 64 in Reference [68]; Figure 12). Compounds were described as having modest potency (<1 µM) in vitro and were shown to be effective in models of thermal injury, the formalin model, Carrageenan, and CFA. An additional patent [63] discloses FTP-THQ that we have assessed above in a number of in vitro and in vivo assays (Figure 6, Figure 7 and Figure 8).

### 5.3. Abbvie Inc.

**Figure 13:**
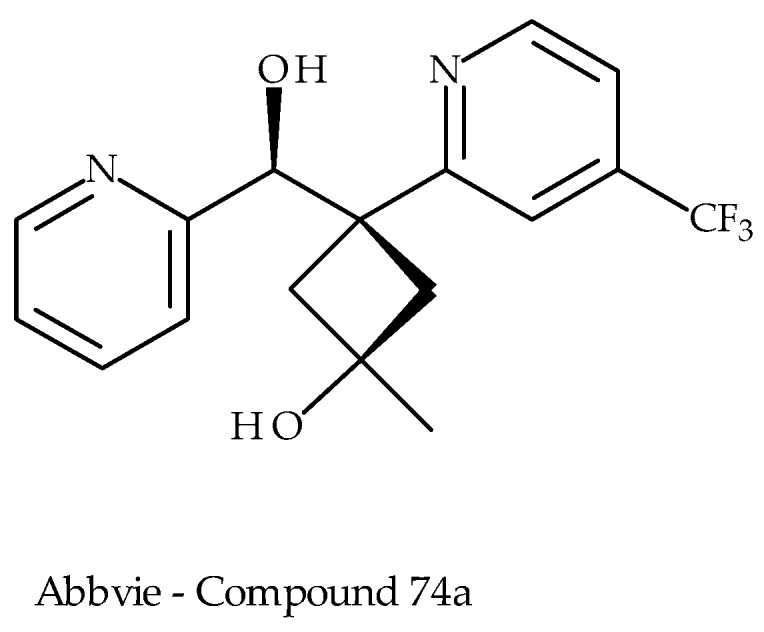
A leading representative from Abbvie’s TRPV3 antagonist series.

In recent years, AbbVie have been active in the TRPV3 arena, publishing several patents [85,86] and more recently a paper [67]. They describe a series of compounds, some displaying mid-nanomolar potency in blocking 2-APB-stimulated calcium influx in assays using recombinant human and mouse TRPV3 channels. Their recent publication describes synthesis and biological properties of a series of (Pyridin-2-yl)methanol derivatives, with the lead molecule (Figure 13) having modest in vitro potency (Kb = 0.56 µM) and efficacy in rat models of neuropathic pain (CCI and SNL).

### 5.4. Miscellaneous

Interestingly, there are also several patents detailing the utility of TRPV3 agonists. One describes derivatives of incensole acetate for use as antidepressants [37,87]. Another details TRPV3 agonists for the treatment of TRPV3-associated skin conditions such as acne, psoriasis, dermatitis, and wound healing, and describes the TRPV3 activity of peptides derived from soricidin (a fifty-four amino acid paralytic peptide isolated from the submaxilary saliva gland of the Northern Short-tailed Shrew, *Blanina brevieauda* [88]).

## 6. Conclusions and Outlook

In summary, we are making headway in understanding the role of TRPV3 in health and disease and this appears to be a promising therapeutic target for itch and skin-related conditions and for the treatment of patients with TRPV3 causally related conditions, such as Olmsted syndrome. To date information from gain of function mutations in rodent and man, coupled with data from gene knock out studies do not provide an overwhelmingly compelling case for targeting TRPV3 as a pain therapeutic; however, pre-clinical data utilizing structurally diverse TRPV3 antagonists do provide some support that blockade of this channel may provide analgesia in chronic pain states. Questions remain however as to how much clinical efficacy for pain can be realized through selectively targeting this mechanism and how results from rodents will translate to humans given the differences in expression in the pain pathway. Given the issues with the side effects associated with targeting other TRP family members, such as TRPV1, including body temperature changes and altered thermal sensitivity, there may be an opportunity to develop topical treatments for TRPV3 given the compelling role in skin.

## Figures and Tables

**Figure 1:**
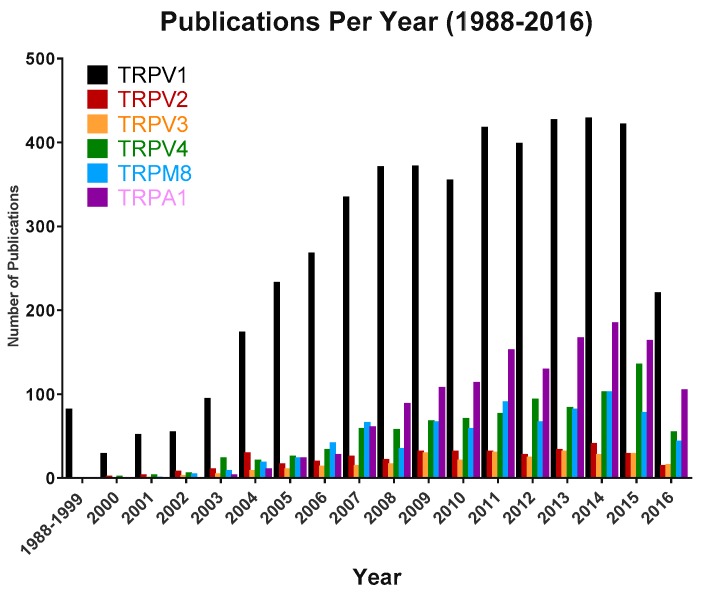
Total publications per year around TRPV3 (orange bars) relative to other thermo-TRPs (TRPV1, TRPV2, TRPV4, TRPM8 and TRPA1). Data as of 25/05/2016. Searches were conducted using PubMed and, where applicable, included alternative nomenclature (e.g., TRPV1 and VR1).

**Table 1:** Summary of Physiological and Pathophysiological roles of TRPV3.

Potential Roles	Summary of Evidence	Reference(s)
Olmsted Syndrome	Several independent clinical reports identify mutations in the TRPV3 gene as a cause of gain-of-function mutations and recessive Olmsted’s Syndrome. Characteristic features include palmoplantar keratoderma, periorificial hyperkeratotic lesions and alopecia. Less common presentations include digit constriction, onychodystrophy and pruritus	[66,69,70,71,72,73]
Olmsted Syndrome with Erythromelalgia	Clinical data of three patients whose disease presentation included intense flares of inflammation, itching, burning pain, vasodilatation, and redness of the extremities consistent with erythromelalgia. Whole exome sequencing identified a de novo heterozygous missense mutation within TRPV3, p.Leu673Phe.	[74,75]
Pruitic and Atopic Dermatitis	Clinical data suggest TRPV3 expression is increased in lesional skin in patients with atopic dermatitis. Preclinical data suggest DS-Nh mice develop allergic and pruritic dermatitis	[58,76]
Psoriasis	Clinical data suggest TRPV3 expression is significantly increased in psoriatic lesions, and that these channels are functional in keratinocytes isolated from lesioned skin. A novel antagonist of TRPV3 dose-dependently inhibited 2APB/carvacrol induced IL-1α release keratinocytes. Similarly, inhibition of EGFR signaling was observed with the antagonist. Inhibitors of both IL-1α release and EGFR signaling have previously attenuated psoriatic symptoms and thus a linkage with TRPV3 is suggested	[55]
Wound Healing	Pre-clinical data suggest higher expression of TRPV3 in mouse oral epithelia versus skin, and expression was upregulated in wounded oral epithelial tissue. TRPV3 activation promoted oral epithelial cell proliferation, which was diminished in TRPV3 knockout mice. Subsequent knock out profiling in a molar tooth extraction model suggest oral wound closure was delayed	[33]
Burn/Post-burn pruritus	Clinical data suggest increased TRPV3 expression in the epidermis of burn scars with pruritus	[29,53]
Hair growth	Pre-clinical data suggest TRPV3 agonists eugenol and 2-aminoethoxydiphenyl borate inhibited hair shaft elongation, suppressed proliferation, and induced apoptosis in human organ-cultured hair follicles. Similarly, functional effects of TRPV3 activation in human ORS keratinocytes were demonstrated as on-target via siRNA	[47]
Skin Barrier Formation	Pre-clinical data suggest TRPV3 forms a direct complex with transglutaminases, thereby regulating growth factor signaling for the formation of the skin barrier	[45]
Rosacea	Clinical data suggest increased TRPV3 expression in epidermal keratinocytes, and dermal labeling was observed in a subset of immune cells and fibroblasts in erythematotelangiectatic rosacea and phymatous rosacea-affected skin. Increased gene expression was also observed in patients with phymatous rosacea	[54]
Cerebral Ischemia	Pre-clinical data suggest the TRPV3 agonist incensole acetate protects against ischemic neuronal damage and reperfusion injury in mice. Reduced infarct volumes, inhibition of TNF-α, IL-1β and TGF-β expression, and NF-κB activation were demonstrated as on-target using TRPV3 knock-out mice	[77,78]
Mastalgia	Clinical data suggest increased expression of TRPV3 in basal keratinocytes that correlated with disease score	[56]
Traumatic Peripheral Nerve Injury	Clinical data suggest increased TRPV3 expression in the DRG neurons of patients with DRG avulsion injury, in the peripheral nerve proximal to the site of brachial plexus injury. However, a decrease in TRPV3 expression was observed in the skin of patients with diabetic neuropathy	[3,79]
Cold- and Heat-evoked Pain	Pre-clinical data showed TRPV3 knockout mice have impaired responses to noxious heat. WBN/Kob-Ht rats, which have a TRPV3 gain-of-function mutation, showed an increased sensitivity to noxious heat and cold stimuli. Multiple antagonists have shown efficacy in inflammatory insult-induced hypersensitivity and nerve ligation-induced hypersensitivity	[45,60,64,65]

## Supplementary Materials

The following are available online at http://www.mdpi.com/1424-8247/9/3/55/s1, Figure S1: The data used for the analyses described in this manuscript were obtained from: the GTEx Portal on 25 May 2016.
