# Supplementary material for: TRPV3 in Drug Development
Source: Pharmaceuticals (Basel). 2016 Sep 9;9(3):55. doi: 10.3390/ph9030055 (PMC5039508; doi:10.3390/ph9030055)
Supplement: Supplementary file 1 [file pharmaceuticals-09-00055-s001.pdf]

Supplementary Materials: TRPV3 in Drug Development

Lisa M. Broad [1,*], Adrian J. Mogg [1], Elizabeth Eberle [2], Marcia Tolley [2], Dominic L. Li [3] and Kelly L. Knopp [3]

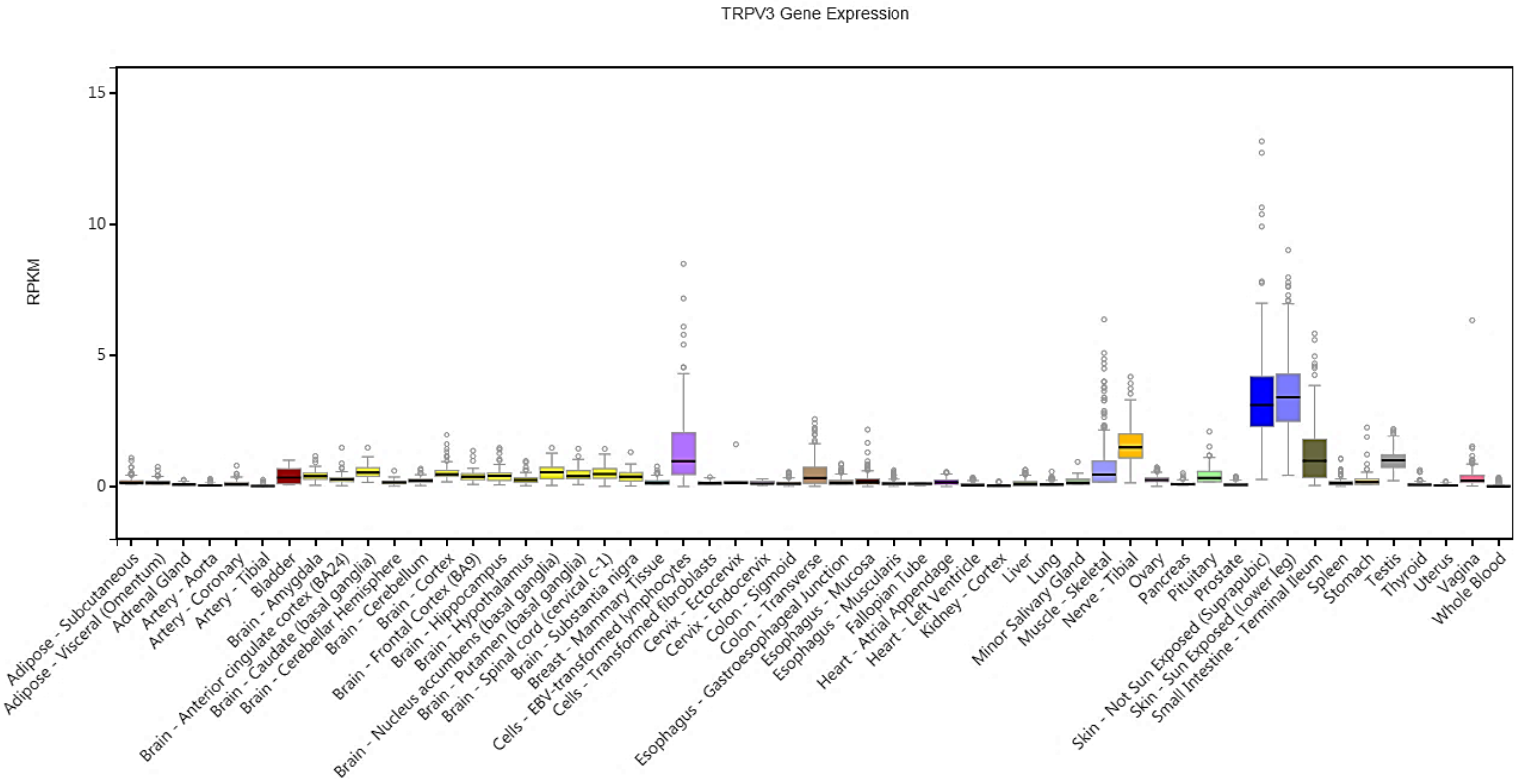

Figure S1. The data used for the analyses described in this manuscript were obtained from: the GTEx Portal on 25/05/16. The Genotype-Tissue Expression (GTEx) Project was supported by the Common Fund of the Office of the Director of the National Institutes of Health. Additional funds were provided by the NCI, NHGRI, NHLBI, NIDA, NIMH, and NINDS. Donors were enrolled at Biospecimen Source Sites funded by NCI/SAIC-Frederick, Inc. (SAIC-F) subcontracts to the National Disease Research Interchange (10XS170), Roswell Park Cancer Institute (10XS171), and Science Care, Inc. (X10S172). The Laboratory, Data Analysis, and Coordinating Center (LDACC) was funded through a contract (HHSN268201000029C) to The Broad Institute, Inc. Biorepository operations were funded through an SAIC-F subcontract to Van Andel Institute (10ST1035). Additional data repository and project management were provided by SAIC-F (HHSN261200800001E). The Brain Bank was supported by a supplement to University of Miami grants DA006227 and DA033684 and to contract N01MH000028. Statistical Methods development grants were made to the University of Geneva (MH090941 and MH101814), the University of Chicago (MH090951, MH090937, MH101820, MH101825), the University of North Carolina–Chapel Hill (MH090936 and MH101819), Harvard University (MH090948), Stanford University (MH101782), Washington University St Louis (MH101810), and the University of Pennsylvania (MH101822).

Supplementary Methods

1.1. Cell Culture

Mouse 308 keratinocytes (m308k) were obtained from the National Cancer Institute (Bethesda, MD, USA) and were grown in EMEM (Lonza, Verviers, Belgium), 10% FBS, and 10% lipumin. All cells were maintained in a humidified 37 °C/5% CO_2 incubator. A day before conducting release experiments, cells were seeded onto 96-well, black-walled, transparent bottom, poly-D-lysine coated plates (BioCoat, Corning, New York, USA) at a density of 20,000 cells per well.

1.2. ATP and GM-CSF Release from Mouse Keratinocytes

On the day of assay growth media was removed from cells plated in 96-well plates, before addition of 50 µl Hanks' Balanced Salt Solution; HBSS (composition, in mM, 138 NaCl, 5 KCl, 0.5 $CaCl_2$, 0.9 $MgCl_2$, 5.6 glucose, 20 HEPES, pH 7.4 adjusted with 5M NaOH) in the presence or absence of antagonists.

For ATP release plates were incubated with antagonists for 10 min at 37 °C. Agonists were then added (50 µL/well) and the plates returned to the incubator for a further 10 min at 37 °C. Finally, the plates were removed from the incubator and 50 µL from each well transferred to a 96-well white Optiplate. ATP release was measured using the ATP Assay System Bioluminescence Detection Kit for ATP Measurement (Promega, Cat No. FF2021, Madison, WI, USA) according to the manufacturer's instructions using a Viewlux (Perkin-Elmer) to quantify the resulting luminescence.

For GM-CSF release plates were incubated with antagonists for 10 min at 37 °C. Histamine (100 µM final concentration) +/− antagonists was then added (50 µL/well) and the plates returned to the incubator for a further 48 h at 37 °C. Finally, the plates were removed from the incubator and 100 µL from each well transferred to a GM-CSF ELISA Kit (Abcam—Cat. No. 46078, Cambridge, UK. The ELISA was run according to the manufacturer's instructions using a Spectramax plate reader (Molecular Devices) to quantify the resulting absorbance at 450 nm.

1.3. Animals

Male Sprague Dawley (SD) rats (body weight range 150 to 200 g) or male CD-1 mice (body weight range 25–28 g) were received from Harlan Laboratories, (Indianapolis, IN, USA). The animals were allowed to acclimate 3 days prior to performing any experimental procedures and were housed 3–4 per cage and allowed standard chow and house water ad libitum. The animals were maintained in a controlled environment with 12 h light cycles (6:00 a.m. to 6:00 p.m.). All experiments were carried out according to protocols approved by the Eli Lilly Institutional Animal Care and Use Committee.

1.4. Mouse Histamine-Induced Itch Model

Harlan CD-1 mice, 4–5 weeks old were acclimated to testing room for 1 h. FTP-THQ was administered at 30, 100, or 200 mg/kg i.p., 1 h prior to histamine, while diphenhydramine was administered at 20 mg/kg, 30 min prior to histamine. Histamine dihydrochloride; (Fluka 53300-1G) was prepared as 2 mg/mL (base) in saline and diluted to 10 µg/25 µL for intradermal injections between the shoulder blades. Animals were then placed inside a clear plexiglass chamber and the number of scratching bouts was scored for 20 min. Data were collected via Abacus software; one-way ANOVA with post-hoc Dunnett's was used for analysis. * $p < 0.05$. Data are expressed as means + SEM, with 8 animals per treatment group unless otherwise specified.

1.5. Rat Formalin Model

The method used for these studies is a modification of that used by Jett et al., 1996. Thirty minutes prior to the administration of formalin, rats were individually placed in cylinders (i.d. 8.5 cm; length 16 cm) on the recording equipment for acclimation. After acclimation in the cylinders, the animals were removed, administered formalin (50 µL of a 5% solution in saline) subcutaneously into the plantar surface of the right hindpaw using a 27½ gauge needle, and immediately placed back into the cylinders. The cylinders were positioned inside of automated test chambers for detecting movement by means of an accelerometer (SR-Lab Startle Response System, San Diego Instruments, San Diego, CA, USA). Rats were monitored continuously for 50 min in 1 s bins. The number of events was defined as the number of 1-s bins with a change in dynamic force that exceeded an empirically determined threshold value (a value of arbitrary load units, which corresponds visually with animals quietly breathing or sniffing). The formalin-induced movements detected by the system included licking and flinching of the affected paw, as well as hopping and turning. The number of "agitation" events was collected and summarized according to the typical biphasic response pattern (Early and Late Phases) in 5-min intervals. After the 50 min test period, animals were sacrificed with CO_2 followed by cervical dislocation. Data was analyzed using One-way ANOVA, and post-hoc analysis of treatment groups compared to the vehicle group using Dunnett's test using JMP statistical software (SAS Institute Inc, Cary, NC, USA). Data are expressed as means ± SEM, with 8 animals per treatment group unless otherwise specified.

© 2016 by the authors. Submitted for possible open access publication under the terms and conditions of the Creative Commons Attribution (CC-BY) license (http://creativecommons.org/licenses/by/4.0/).
